# Periodontal Breakdown, Orthodontic Movements and Pulpal Ischemia Correlations—A Comparison Between Five Study Methods

**DOI:** 10.3390/jcm13237062

**Published:** 2024-11-22

**Authors:** Radu-Andrei Moga, Cristian Doru Olteanu, Ada Gabriela Delean

**Affiliations:** 1 Department of Cariology, Endodontics and Oral Pathology, School of Dental Medicine, University of Medicine and Pharmacy Iuliu Hatieganu, Str. Motilor 33, 400001 Cluj-Napoca, Romania; ada.delean@umfcluj.ro; 2 Department of Orthodontics, School of Dental Medicine, University of Medicine and Pharmacy Iuliu Hatieganu, Str. Avram Iancu 31, 400083 Cluj-Napoca, Romania

**Keywords:** periodontal breakdown, dental pulp, neuro-vascular bundle, orthodontic movements, numerical methods, finite elements analysis

## Abstract

**Background/Objectives**: This study assessed the biomechanical behavior of dental pulp and the neuro-vascular bundle/NVB as well as the ischemic risks during orthodontic movements in a gradual horizontal periodontal breakdown, using five methods and aiming to identify the most accurate one. **Methods**: Seventy-two models of second lower premolar (from nine patients) were subjected to 3 N of intrusion, extrusion, rotation, tipping, and translation. Five numerical methods, Tresca, Von Mises/VM, Maximum and Minimum Principal, and hydrostatic pressure were used in a total of 1800 numerical simulations. The results were color-coded projections of the stress areas that were then correlated with maximum physiological hydrostatic pressure/MHP and known clinical biomechanical behavior. **Results**: During periodontal breakdown, all five methods displayed, for all movements, quantitative stresses lower than MHP, suggesting that 3 N are not inducing any local tissular ischemic risks for the healthy intact tissues. All five methods displayed rotation as the most stressful movement during periodontal breakdown, while translation was the least. The NVB was more exposed to ischemic risks than dental pulp during the periodontal breakdown due to constant tissular deformations. Only VM and Tresca methods showed translation as more prone to expose dental pulp (both coronal and radicular) to ischemic risks (than the other movements) during the periodontal breakdown simulation. However, all five methods showed intrusion and extrusion as more prone to expose the NVB to higher ischemic risks than the other movements during the periodontal breakdown simulation. **Conclusions**: During periodontal breakdown, Tresca and Von Mises were more accurate, with Tresca being the most accurate of all.

## 1. Introduction

There is a continuous, increasing interest in the individualized study of each dental tissular component, manifested through new numerical studies [1,2,3,4]. Dental pulp and the neuro-vascular bundle/NVB are one of the least studied among the dental tissues despite their great importance during orthodontic treatment [5,6,7,8,9,10,11,12,13]. Under orthodontic loadings, various amounts of local circulatory disturbances appear, triggering the orthodontic movements [5,6,7,8,9,10,11,12,13,14]. The physiological tissular local maximal hydrostatic pressure/MHP is 16–22 KPa (about 80% of the systolic pressure) [15,16,17,18,19,20,21,22]. Any pressure exceeding this, induced by the orthodontic loads, will lead to a variable degree of changes in the pressure amount, with a partial collapse of circulatory vessels and localized ischemia (triggering the orthodontic movement) [23,24,25,26,27,28,29,30,31,32,33]. However, if the amount of force is too high or applied for longer periods of time it will locally induce prolonged ischemia followed by necrosis and degenerative-resorptive mechanisms [23,24,25,26,27,28,29,30,31,32,33]. Despite being clinically accepted that light orthodontic forces of up to 1 N are safe for the intact periodontium [15,16,17,18,34], there are multiple studies regarding the optimal amount of force for intact periodontium, with various contradicting results, reporting variable 1–3 N and various movements as more tissular stressful (e.g., rotation vs. intrusion), creating more confusion [35,36,37,38,39,40] and no consensus. However, there is no available data regarding the amount of force safely applied in various amounts of reduced periodontium, despite being clinically quite frequent.

It must be emphasized that healthy intact tissues hold the ability to adapt to these local changes and naturally avoid any necrosis or resorptive processes [23,24,25,26,27,28,29,30,31,32,33]. However, if the living tissues were previously traumatized/injured (e.g., occlusal trauma for the NVB [9,10,11,12,13] and direct/indirect pulp capping for dental pulp [23,24,25,26,27,28,29,30,31,32,33]), some morphological structural changes occur with diminishing the tissular adaptability potential [9,10,11,12,13,33,41,42,43,44,45,46,47,48]. Thus, an orthodontic force that in healthy intact tissues will pose no ischemic risks, in a diminished tissular adaptability situation could induce ischemia, followed by necrosis and further tissular loss. Unfortunately, these previous injuries and functional tissular changes are not clinically directly visible (but can be predicted); thus, in the oral cavity only the ischemic results will be seen. Thus, an accurate method to anticipate these ischemic risks is of extreme importance for planning orthodontic treatment and predicting its outcome.

Moreover, the biomechanical tissular individual behavior is variable at various levels of periodontal breakdown [9,10,11,36,37,39,40,42,43,49,50,51,52,53,54,55,56,57,58,59,60,61,62,63,64] with great influence over the tissular stress distribution and directly influencing the localized circulatory disturbances in pulp and the NVB. Most of the orthodontic patients have various amounts of bone loss (usually of a few mm), with direct influence over the amount and local orthodontic stress distribution. There are no studies to be found regarding the biomechanical behavior of dental pulp and the NVB during the periodontal breakdown. Moreover, there are no comparative studies about the accuracy of study methods. We previously approached some of these above-mentioned issues but only for the light orthodontic forces, but no data are available for larger forces [15,16,17,18,19,20,21,22].

Clinical studies are not able to individually study each dental component but can provide a general picture of the biomechanical behavior of the entire tissular structure (i.e., tooth with pulp, the NVB, periodontal ligament and bone) [15,16,17,18,19,20,21,22]. The only method enabling the individual analysis of each structure is the numerical method based on radiological CBCT images (cone beam computed tomography) [1,2,3,4]. Nevertheless, due to the small dimensions of these dental tissues (pulp and the NVB), the radiological image must have a small voxel size of less than 0.01 mm [15,16,17,18,19,20,21,22].

The numerical methods used in dental studies were originally designed for the engineering field, where they are still extremely accurate and reliable (and widely used) [64]. However, since they were imported into the dental field, their accuracy was dramatically reduced (even for the new studies as well [1,2,3,4]), providing reports that often contradicted the clinical data, varying from one report to another and creating debatable results and a mistrust of the method [15,16,17,18,19,20,21,22]. The most studied were the PDL and dental implant–bone interfaces, while the newer studies are extended to the entire oral cavity [1,2,3,4]. These accuracy issues (never addressed and resolved) were directly linked (as reported by our previous reports [15,16,17,18,19,20,21,22]) to the misunderstanding of the numerical studies methodological protocol. The numerical study employs a certain methodology based on the analyzed type of material (brittle, ductile and liquid/gas), an extremely accurate model, as well as proper boundary assumptions [15,16,17,18,19,20,21,22,64]. None of these were approached in the current numerical dental studies research flow [15,16,17,18,19,20,21,22,64]. Most of the studies [35,36,37,38,39,40,49,50,51,52,53,54,56,57,58,59,60,61,62,63,64,65,66] employed (still continuing the same pattern [1,2,3,4]) a numerical method without discussing if it is suitable for the investigated tissues [15,16,17,18,19,20,21,22]. Moreover, correlations with the physiological MHP values are rarely seen [15,16,17,18,19,20,21,22]. The still employed numerical methods [35,36,37,38,39,40,49,50,51,52,53,54,56,57,58,59,60,61,62,63,64,65,66] are Maximum (tensile) and Minimum (compressive) Principal, Von Mises/VM (overall), Tresca (shear, only recently introduced) and hydrostatic pressure (liquids/gas), despite the general acceptance that dental tissues are of ductile resemblance with a certain brittle flow mode [15,16,17,18,19,20,21,22]. Thus, the reported results contained multiple inconsistencies [35,36,37,38,39,40] that contradicted the clinical reality [5,6,7,8]. In previous research, Tresca along with Von Mises was reported for light orthodontic forces to be the most accurate [15,16,17,18,19,20,21,22]. No similar data are available for large forces and various levels of bone loss [15,16,17,18,19,20,21,22]. Additionally, some concerns were also raised due to reports regarding the poor quality of multiple in vivo studies [5,7,42] used for indirect validation.

Since numerical methods are the only possibility to analyze dental tissue [15,16,17,18,19,20,21,22,64] (e.g., pulp and NVB structures), an accurate working method is needed, but only based on a scientific comparison between multiple methods.

Our aim was to assess the biomechanical behavior of dental pulp and the neuro-vascular bundle as well as the induced ischemic risks during five orthodontic movements and 3 N in a gradual horizontal periodontal breakdown of 1–8 mm, using five numerical methods. Additionally, another objective was to analyze the differences between the reports of these five numerical methods and to identify the most accurate of all in the study of pulp and the NVB.

## 2. Materials and Methods

This study is part of a larger research project conducted in a stepwise manner (with clinical protocol 158/02.04.2018) and studying the biomechanical behavior of dental tissues in intact and reduced periodontium [15,16,17,18,19,20,21,22]. This study focused on assessing the most suitable method of study to obtain the most accurate results.

Our study involved nine patients (4 males/5 females, mean age 29.81 ± 1.45) and totaled 1800 numerical simulations. The simulations were performed on 72 3D models of a healthy intact second lower premolar.

The inclusion criteria were as follows: no malposition and missing teeth in the investigated region, intact healthy second lower premolar, limited to 1–2 mm bone loss around the second lower premolar, orthodontic treatment indication and non-inflamed periodontium associated with proper oral hygiene. The exclusion criteria were as follows: more than 2 mm bone loss, particular root geometry, various crown and root abnormalities, radiologically visible bone defects and poor oral hygiene after inclusion.

**Figure 1:**
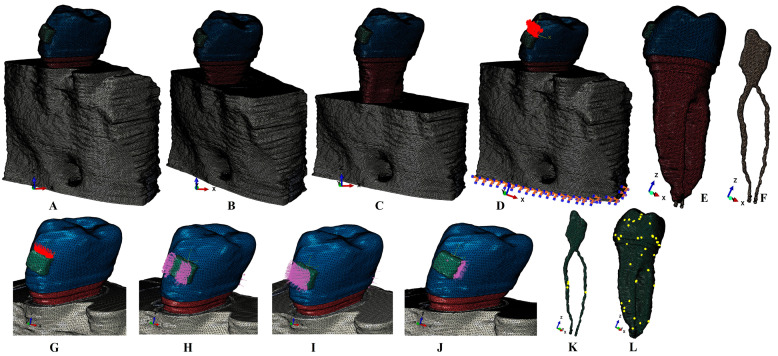
Mesh model (one of the nine 3D models): (**A**) 2nd lower right premolar model with 1 mm loss periodontium, (**B**) 4 mm loss model, (**C**) 8 mm loss model, (**D**) 1 mm loss models with applied extrusion and restraining movements base vectors, (**E**) second lower premolar with the base of the bracket and the NVB and (**F**) dental pulp and NVB. Applied vectors: (**G**) intrusion, (**H**) rotation, (**I**) tipping, (**J**) translation, (**K**) dental pulp and NVB mesh with elements warnings and (**L**) second lower premolar with mesh elements warnings.

The region of interest held the lower first molar and two premolars and was radiologically investigated by a CBCT (cone beam computed tomography—ProMax 3DS, Planmeca, Helsinki, Finland; voxel size of 0.075 mm). For manual tissular reconstructions, the AMIRA 5.4.0 (Visage Imaging Inc., Andover, MA, USA) was employed. The manual reconstruction process identified on DICOM images all tissular components (enamel, dentine, dental pulp, the neuro-vascular bundle, the periodontal ligament/PDL and trabecular and cortical bone). All above-mentioned tissues were assembled into 3D models (Figure 1). The PDL had a physiological variable thickness of 0.15–0.225 mm. The cementum component could not be properly separated from dentine; thus, based on the physical similarity between their properties, cementum was reconstructed as dentine (Table 1). The molar and the first premolar were not reconstructed, while their alveolar sockets were filled with trabecular and cortical bone. The missing bone and PDL were reconstructed for obtaining intact periodontium models. A base of a stainless-steel bracket was reconstructed on the vestibular side of the enamel.

For obtaining a gradual horizontal periodontal breakdown, each of the nine intact periodontium models were subjected to a gradual loss of 1 mm, obtaining thus various bone loss models (1–8 mm loss).

The mesh models had 5.06–6.05 million C3D4 tetrahedral elements, 0.97–1.07 million nodes and global element size of 0.08–0.116 mm (Figure 1). There were no mesh errors, but only a reduced number of element warnings (e.g., for one of the models: pulp–NVB mesh with 4 element warnings, meaning 0.0158% for a total of 25,252 elements—Figure 1K, and tooth mesh with 39 element warnings, meaning 0.00589% for a total of 661,137 elements—Figure 1L). A mesh element warning means a small non-essential areas discontinuity, but with no impact over the biomechanical behavior of the model.

The numerical analysis employed the ABAQUS 6.13-1 (Dassault Systèmes Simulia Corp., Maastricht, The Netherlands) and used the following five most used numerical methods/failure criteria in dental studies: Von Mises (maximum overall), Tresca (maximum shear), Maximum Principal (maximum tensile), Minimum Principal (maximum compressive) and hydrostatic pressure (liquids/gas). As boundary assumptions, the base of the models had zero displacements, and isotropy, linearity, and homogeneity/non-homogeneity (as in most of the current numerical studies). Five orthodontic movements were simulated: extrusion, intrusion, rotation, tipping and translation and an orthodontic force of 3 N. The results were displayed as color-coded projections of various intensities (red-orange for high, yellow-green for moderate and blue for low) showing the tissular deformations induced by the orthodontic movements. Correlations with the maximum physiological hydrostatic pressure of 16–22 KPa as well as with the known clinical biomechanical behavior were performed to assess the ischemic risks.

## 3. Results

**Figure 2:**
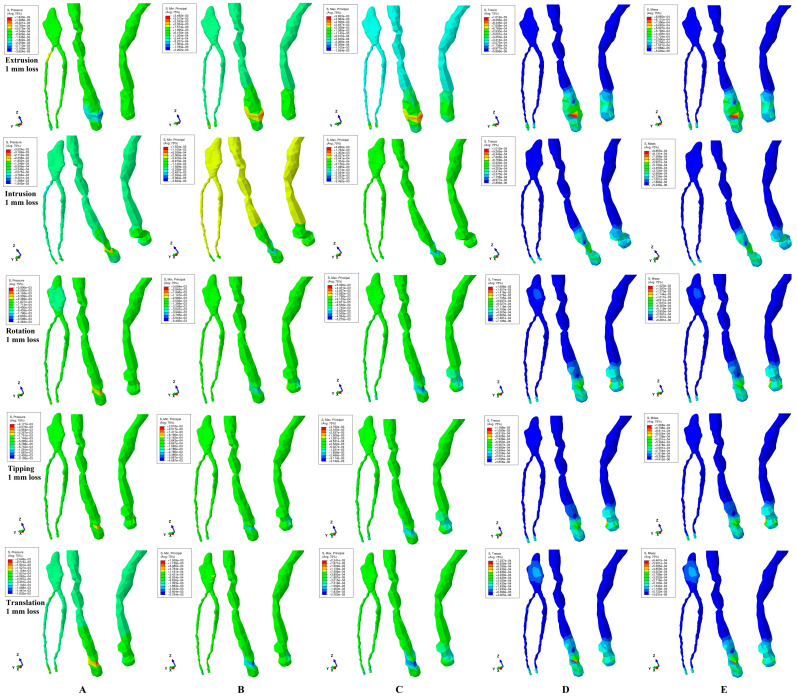
Comparative stress distribution displayed by the five methods for 3 N applied force (of one of the nine 3D models), in 1 mm periodontal breakdown and five movements (extrusion, intrusion, rotation, tipping and translation): (**A**) hydrostatic pressure, (**B**) Minimum Principal, (**C**) Maximum Principal, (**D**) Tresca and (**E**) Von Mises.

**Figure 3:**
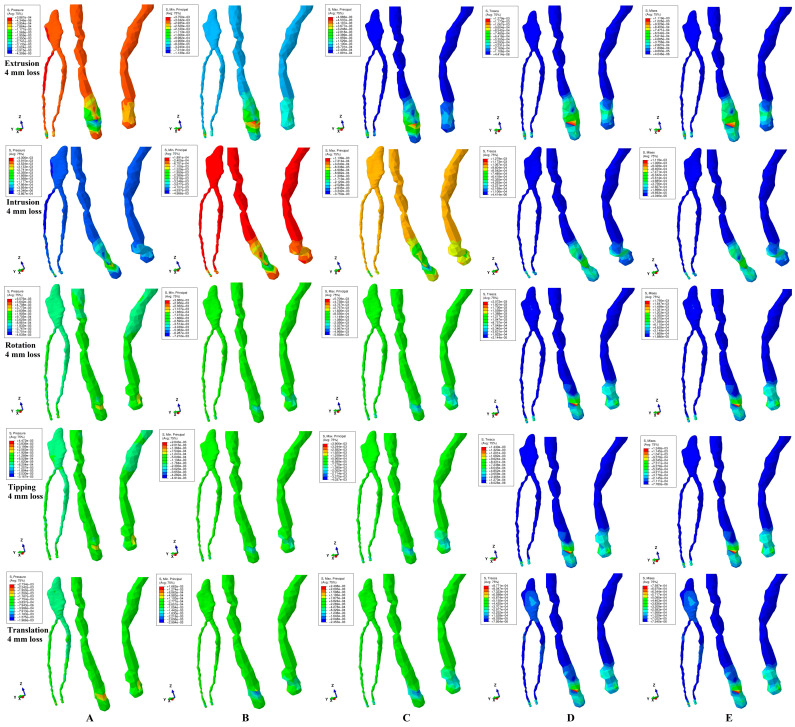
Comparative stress distribution displayed by the five methods for 3 N applied force (of one of the nine 3D models), in 4 mm periodontal breakdown and five movements (extrusion, intrusion, rotation, tipping and translation): (**A**) hydrostatic pressure, (**B**) Minimum Principal, (**C**) Maximum Principal, (**D**) Tresca, (**E**) Von Mises.

**Figure 4:**
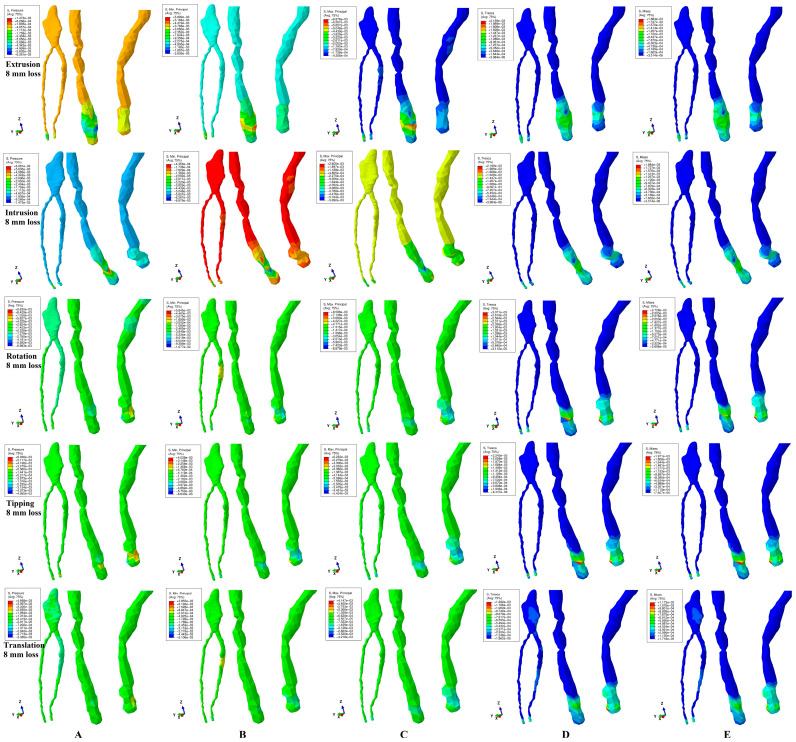
Comparative stress distribution displayed by the five methods for 3 N applied force (of one of the nine 3D models), in 8 mm periodontal breakdown and five movements (extrusion, intrusion, rotation, tipping and translation): (**A**) hydrostatic pressure, (**B**) Minimum Principal, (**C**) Maximum Principal, (**D**) Tresca and (**E**) Von Mises.

All five methods analyzing the pulp and NVB biomechanical behavior (Figure 2, Figure 3 and Figure 4 and Table 2) during the five orthodontic movements and gradual horizontal periodontal breakdown showed that the maximum amount of stress was present in the NVB, while the pulpal stress was extremely small. Thus, the NVB stress was ranging between 1.17 and 8.87 KPa, while the pulpal stress was of 0.16–0.48 KPa, for 8 mm bone loss (Table 2), lower than the 16–22 KPa of the local maximum physiological hydrostatic pressure.

It seems that 3 N of force induces no ischemic risks during orthodontic movements and periodontal breakdown (up to 8 mm tissular loss) for healthy intact teeth. However, since the NVB stress was 7.3–18.47 times higher than the pupal stress (for 8 mm loss), it is more exposed to ischemic risks than the pulp. The highest amount of stress was present (in each of the five methods) at 8 mm of loss, showing that the periodontal breakdown induces a direct increase in tissular stress (especially visible in the NVB).

Rotation movements (in each of the five methods) displayed the highest amount of stress (Table 2), seeming to be the most stressful movement, closely followed by tipping and intrusion/extrusion, while the translation was the least of them.

The hydrostatic pressure method/failure criteria displayed the highest amounts of stress, closely followed by the Maximum (tensile) and Minimum (compressive) Principal, while the Von Mises (overall) and Tresca (shear) were the least of them.

In all movements and bone loss levels, the coronal and radicular pulp displayed similar qualitative stress (with two exceptions for rotation and translation for VM and Tresca methods), suggesting that the orthodontic force induced limited tissular stress.

The VM and Tresca methods are the only methods to display localized areas of coronal pulpal stress (more pronounced for translation) (Figure 2, Figure 3 and Figure 4). For the 1 mm bone loss level, during translation, the coronal pulpal stress displayed the highest spread on vestibular, mesial and distal sides of coronal pulp (Figure 2D,E) but with no radicular pulp stress. At a 4 mm loss, the stress areas extend to the radicular pulp (Figure 3D,E) in the cervical and middle thirds of the root. An 8 mm periodontal loss displayed limited coronal pulpal stress (Figure 4D,E), with an increase in the middle third of the root (correlated with the bone loss level). The rotation movement displayed a localized reduced coronal pulpal stress at the 1 mm periodontal breakdown, completely disappearing at 4 mm loss. Based on the above, despite being the least stressful among the five movements (safe for healthy intact tissues), the translation seems to directly influence the coronal pulp, inducing potential ischemic risks at this level (especially on previously injured tissues—direct/indirect pulp capping).

Quantitatively, all five methods displayed a similar pattern of increased stress (Table 2), the only difference being their amounts. Thus, hydrostatic pressure and Maximum and Minimum Principal methods displayed quantitative results 7–18 times higher than Von Mises and Tresca, but with no possible scientific evaluation of their accuracy (since all were lower than MHP).

However, qualitatively, there are significant differences between their biomechanical behavior stress displays, which can provide an orientation to their accuracy (Figure 2D,E, Figure 3D,E and Figure 4D,E). Thus, Tresca and Von Mises methods/criteria are the only ones to display the same constant stress distribution pattern during the entire periodontal breakdown and for all five movements (constant extremely small intensity blue shades amounts of stress for pulp and various amounts of higher/medium intensity stress for the NVB). This stress display pattern is correct when compared with the known clinical tissular biomechanical behavior, and it is due to the correct design (based on the analyzed type of material) of these two methods for ductile-like materials (dental tissues being materials of ductile resemblance). When comparing the two criteria, another aspect must be addressed, respectively, the homogeneity/non-homogeneity nature of the analyzed material. The Von Mises method was designed for homogenous materials and the Tresca method for non-homogenous materials, while the living dental tissues are non-homogenous materials. Thus, despite qualitatively showing no differences, quantitatively the Tresca amounts of stress are a little bit higher when compared with Von Mises (1.12–1.17 times, falling into the literature average interval of 1.15—Table 2). Based on the above, Tresca seems quantitatively more accurate than Von Mises.

The hydrostatic pressure method/criteria displayed various inconsistencies (Figure 2A, Figure 3A and Figure 4A) in the intensity of stress display with significant variables from one movement to another and from one bone loss level to another. This biomechanical behavioral stress display is due to the failure criteria specially designed for liquids and gas where there are no shear stresses and an internal micro-architecture fundamentally different from that of the studied dental tissues (despite the high percentage of contained water/liquid, which is the main reason for it to be employed in the first place).

The Maximum (tensile) and Minimum (compressive) stress methods/failure criteria displayed the same qualitative inconsistencies (Figure 2B,C, Figure 3B,C and Figure 4B,C) as hydrostatic pressure, with various intensities of color-coded stress displays, different from one movement to another and from one periodontal breakdown level to the other. This behavioral display is because both criteria were specially designed for brittle-like materials while the dental tissues are known to have a ductile resemblance.

These three methods displayed both qualitative biomechanical behavioral pattern inconsistencies (variable mixes of negative–positive stresses, with no scientific explanation, since no compressive-tensile stresses should be present in these enclosed/protected areas) contradicting the physical known structural clinical behavior, as well as higher amounts of stress (when compared to the first two methods), seeming to be less accurate for the study of pulp and the NVB. The similar color-coded stress display in both pulp and the NVB suggests comparable tissular ischemic risks despite the anatomical topography. Biomechanically, the pulp is protected by dentine (pulp chamber and radicular root canals), while the NVB is held in the apical third of the periodontal ligament (directly exposed to stresses). Thus, it seems that the qualitative display contradicts the quantitative display (with the NVB higher than pulpal stress—Table 2) as well as clinical knowledge.

Nevertheless, despite the above-mentioned differences, all five methods similarly displayed the NVB tissular deformations induced by the orthodontic movements during the periodontal breakdown, seeming to provide a correct picture of the clinical biomechanical behavior for larger tissular deformations/effects. Despite all quantitative results being lower than MHP (i.e., 3 N are safely applied in healthy intact tissues), the displayed NVB tissular deformations could have a potential ischemic risk on previously injured tissues (e.g., occlusal trauma).

## 4. Discussion

Despite the importance of the NVB and dental pulp during orthodontic treatment, there is a limited number of numerical studies (some of them belonging to our team [15,16,17,18,19,20,21,22]) with a focus on these tissues, due to segmentation and 3D reconstruction difficulties. Most of these studies are for intact periodontium, and only one focuses on periodontal breakdown [18]. No other study performing a correlation between the five methods addressing the tissular biomechanical behavior issues was found in the current research flow (except ours); thus, this study is the first. However, since the NVB is held in the apical third of the periodontal ligament (absorption-dissipation function [15,16,17,18,19,20,21,22]), correlations with numerical PDL studies as well as with MHP (16–22 KPa) can be made.

Our numerical study assessed the biomechanical behavior of dental pulp and NVB during 1–8 mm periodontal breakdown totaling 1800 numerical simulations. Tresca, Von Mises, Maximum and Minimum Principal and hydrostatic pressure methods were all used by the current dental numerical studies [1,2,3,4,35,36,37,38,39,40,49,50,51,52,53,54,55,56,65,66]. The Tresca method was introduced in the current research flow by our previous research [15,16,17,18,19,20,21,22], in order to develop and provide an accurate method for the study of dental tissues [64].

Each of the five above-mentioned methods have been developed in the engineering field for the study of different types of materials (with acknowledged accuracy), an issue of extreme importance when studying dental tissues [64]. These methods had been specifically designed for a certain type of studied material [64]. The Maximum (tensile) and Minimum (compressive) Principal had been designed for brittle-like materials, that when subjected to loads suffer from non-recoverable form deformations, leading to small deformations and rapidly followed by cracking and destruction (e.g., glass and concrete) [15,16,17,18,19,20,21,22]. The Tresca (shear) and Von Mises (overall) were designed for ductile-like materials, that when subjected to loads suffer recoverable form deformations, totally recovering the form when the load stops (e.g., rubber and steel) [15,16,17,18,19,20,21,22]. Hydrostatic pressure was specially designed for liquids and gas (no shear stress), materials with no internal structure [15,16,17,18,19,20,21,22].

In numerical studies, the employed method is selected based on the analyzed material type (ductile, brittle or liquid/gas) to provide accurate results [64]. Dental tissues are ductile-like materials with a certain degree of brittle flow mode [15,16,17,18,19,20,21,22,64]. None of the previous numerical studies (except ours) had this approach, thus, reporting results with serious accuracy issues. Another issue is the natural non-homogeneity of dental tissues; thus, the employed method must fulfill this request (e.g., only Tresca was specifically designed for non-homogenous materials) [15,16,17,18,19,20,21,22,64]. On the other side, dental studies [1,2,3,4,35,36,37,38,39,40,49,50,51,52,53,54,55,56,65,66] used forces up to 6 N (extremely small when compared with the engineering field), and the tissular deformations and displacements were extremely small during the orthodontic movements; thus, differences when employing one of the five methods are less visible than when displaying the biomechanical results side by side (as in this study). The dental pulp is held in the dental pulp chamber, an incompressible hard tissue cavity made of dentine, with a protection role, where there are virtually no deformations and displacements. On the other hand, the NVB is held in the apical third of the PDL, an extremely deformable and tensile-compressive tissue, with nutritional, metabolic and absorption-dissipation roles. Thus, the NVB is extremely prone to deformations and displacements, within the PDL deformations limits, and subjected to ischemic risks. It must be emphasized that this study is the first to address the benefits of this approach in the current research flow.

When the biomechanical behavioral results of the five methods are displayed side-by-side and compared, some general patterns (in agreement with known clinical data) are similar: the NVB stress is always higher than pulpal stress (quantitatively), while the NVB tissular deformations induced by the movements in various bone loss levels are clearly visible, in agreement with known clinical tissular biomechanics. However, when further investigating the side-by-side qualitative display, the accuracy issues became more and more visible (all related to the misused method) [15,16,17,18,19,20,21,22]. Based only on the quantitative results (all lower than MHP, see Table 2), it is difficult to distinguish a scientifically based difference (although two of the criteria showed very low values compared to the other three—in line with other studies [15,16,17,18,19,20,21,22]).

Qualitatively, Tresca and VM displayed a constant, similar pattern of color-coded blue small intensity stress for dental pulp for all five movements during the periodontal breakdown simulation, which is in agreement with the known tissular clinical biomechanical behavior and other reports [5,7,9,10,11,12,33,34,42,43,44,45,46,47,48,64], as well as our previous research [15,16,17,18,19,20,21,22]. The quantitative values were also the smallest among the five methods, 5.2–145.5 times under MHP, posing no ischemic risks for the 3 N of applied force, which is also in agreement with previous reports and the above-mentioned clinical data. A previous study [18], with similar methodology using 0.5 N of force, reported similar correlations as this study, with VM and Tresca being more accurate, with Tresca better suited for dental tissues. However, translation, despite being the least stressful movement among the five, displayed coronal pulp and radicular stress in both methods (Figure 2D,E, Figure 3D,E and Figure 4D,E) during the entire horizontal periodontal breakdown simulation. Despite being quantitatively lower than MHP, with no ischemic risks for healthy tissues, if pulpal tissues suffered of an earlier injury (i.e., direct/indirect pulp capping [9,10,11,12,33,42,43,44,45,46,47,48]), ischemic risks are expected (in agreement with clinical data [13,41]).

The tissular NVB deformations reported by all five methods, are in line with known clinical biomechanical behavior and other reports [5,7,9,10,11,12,33,34,42,43,44,45,46,47,48,64]. All five methods reported quantitative amounts of stress lower than MHP, thus posing no ischemic risks for healthy intact tissues. However, there are reports of ischemia and necrosis in previously injured tissues (i.e., occlusal trauma [9,10,11,23,24,25,26,27,28,29,30,31,32,33]); thus, our study is in agreement with those studies [23,24,25,26,27,28,29,30,31,32,33]. Generally, all five methods displayed NVB deformations and lower (than MHP) quantitative amounts of stress (despite other visible differences), due to the extremely small tissular biomechanical deformations and displacements imposed by the anatomical micro-architecture. However, if the local anatomical topographic conditions would have allowed larger deformations and tissue displacements, these qualitative–quantitative differences would have been even larger, confirming the above-mentioned.

Rotation was reported to be the most stressful by all five methods during the periodontal breakdown, in agreement with our previous research [15,16,17,18,19,20,21,22], other research [23,24,25,26,27,28,29,30,31,32,33] and reports by Wu et al. [35,36,37]. Intrusion and extrusion were also seen as stressful movements (Table 2), in agreement with reports by Minch et al. [38] and Hofman et al. [39,40] (but for the intact periodontium).

The other three methods (Maximum and Minimum Principal and hydrostatic pressure), despite displaying quantitative data lower than MHP and confirming the safety of 3 N appliance during periodontal breakdown, displayed qualitative stress patterns with various inconsistencies (negative/positive compression-tension in dental pulp where this was not clinically present and bizarre variations in color-coded stress display from one movement to another and between bone loss levels) with no biomechanical scientific explanations. Since the dental pulp is held in an incompressible cavity made entirely of solid material, no deformations or displacements clinically occur, and thus the color-coded projections should display the same color of low/no stress intensity during the entire periodontal breakdown. However, the three methods displayed various stress intensities contradicting the mechanical physical principles. Thus, the only possible explanation is that the lower accuracy is due to design issues (brittle/liquid type of material).

The analysis of other numerical reports [1,2,3,4,35,36,37,38,39,40,49,50,51,52,53,54,55,56,65,66] confirmed the above-mentioned differences. Multiple intact periodontium PDL numerical studies have assessed a few orthodontic movements, one/two [39,40,56] anatomically simplified 3D models (i.e., upper first premolar [36,37,39,40,56]; canine [35,36,37]; first molar [65] and incisor [37,50,51,52,53,54]), intact periodontium [35,36,37,39,40,56] or variable levels of reduced periodontium [52,53,65] and employed failure criterions (hydrostatic pressure [35,36,37,39,40,56,66], Maximum and/or Minimum Principal [49,50,51,52,53] and VM [49,54,56]) without any reference to the type of material studied, homogeneity/non-homogeneity or MHP [1,49,50,51,52,53,54], employing non-linearity [49,50,51]/linearity [52,53,54].

Some reported results exceeded physiological MHP [49,50,51,52,53,66] when using 1–6 N loads [35,36,37,39,40,66], and with hydrostatic pressure [39,40,56] being the only method for the study of PDL (despite not performing any comparison/correlation with other methods), this contradicts clinical knowledge. Wu et al. reported as optimal a rotation of 2.1–2.9 N [35,36,37], contradicting Profit et al. [34] and clinical knowledge (optimal should be light to avoid any ischemic and necrotic risks).

These major issues were due to tissular models since PDL studies [1,35,36,37,38,39,40,49,50,51,52,53,54,56,65,66] did not reconstruct the NVB structure. Thus, despite reporting various amounts of stress, the accuracy is debatable (e.g., the PDL apical third amounts of stress exceeding MHP for light orthodontic forces [35,36,37,39,40] are clinically incorrect). More biomechanically bizarre is the qualitative stress display of extremely high PDL apical third stress associated with insignificant cervical third stress [35,36,37], which is biomechanically fundamentally incorrect [9,10,11,34,42,43,50,51]. From the mechanical physics point of view, correlated with tissular anatomical micro-architecture, the first major stress should be displayed in the cervical third, where the PDL surface is important, and the absorption-dissipation ability is high. Additionally, in the apical third from the same mechanical point of view, the stress should be at a minimum since most of the stresses are absorbed and dissipated in the middle and cervical thirds. Moreover, the vascularization is much better in the apical third than in cervical and middle thirds, while the natural tissular adaptation ability is higher. Other research (with no NVB tissular reconstruction) reported the opposite, with high cervical stress and almost no apical third stress [1]. The above-mentioned reports [1,35,36,37,38,39,40,49,50,51,52,53,54,55,56,65,66] suffer from the same methodological issues: the employed method lacked a material-based method selection and no correlations/comparations with other methods.

The numerical method limits are related to the employed proper failure criteria and the anatomical accuracy of the 3D model, as well as multiple simulations on the same model for indirect validation. Due to the importance of pulp and NVB functionality in orthodontic treatment [5,6,7,8,9,10,11,12,13,14], and the need for an individualized biomechanical behavioral study, numerical studies are the only option. Numerical studies are not able to completely mimic the clinical conditions (usually there are no pure movements and only associations). Thus, clinically, the quantitative amounts of stress could be a little bit lower than in this study, but with no impact over the report’s accuracy.

The proper method/failure criteria selection is related to the brittleness–ductileness of the analyzed tissues [64]. There are many new numerical studies employing brittle-like material methods for the dental tissues of ductile resemblance (i.e., the same as the above-mentioned flows) reporting results with accuracy issues [1,2,3,4]. In the engineering field, there are significant mechanical behavioral differences between brittle and ductile materials. In dentistry, due to limited deformations and displacements, these differences are seen only if the behavioral results are put side by side. Nevertheless, the differences are significant from the mechanical point of view when describing the tissular biomechanics. The anatomical accuracy of the models is directly related to the number of elements and nodes and the small global element size (i.e., a variable PDL thickness of 0.15–0.225 mm vs. a constant thickness of 0.25 mm [1]; 6 million tetrahedral elements and 1 million nodes, global element size of 0.08–0.116 mm, 40–12,731 times more elements, 4.4–1463 times more nodes than previous studies [9,10,11,39,40,42,43,49,50,51,52,53,54,55,56]). The CBCT image accuracy (small voxel size) also influences the anatomical accuracy (a voxel size of 0.075 mm herein vs. 0.3 mm [1]). A larger sample size also enhances the result accuracy (nine in this study vs. one in current studies [1,50,51,54,57,58,59,60,61,62,63,64]). Multiple simulations also increase the reports’ accuracy (1800 in this study vs. a few in most of the other studies [35,36,37,38,39,40,49,50,51,52,53,54,56,57,58,59,60,61,62,63,64,65,66]). Since there are so many numerical studies with accuracy issues (due to a methodological lack of understanding) [5,7,42,64], there is a need for numerical studies to address the above-mentioned issues. The limitations of the numerical studies in terms of clinical practice are mostly related to the fact that they cannot accurately reproduce the clinical situations, meaning that the quantitative results herein provided are lower than clinical ones. Qualitatively, since the associations of movements are often used (instead of pure movements as in this study), this means that the stress display areas are in clinical reality merged. Nevertheless, the results from this study do not lose their accuracy, since each time the results are clinically lower, while this study provided the maximum amount of stress and tissular deformations induced by the movements.

## 5. Conclusions

1. During the periodontal breakdown, all five methods displayed, for all five movements, quantitative amounts of stress lower than MHP, suggesting that 3 N are not inducing any local tissular ischemic risks for the healthy intact NVB and pulp.
2. During the periodontal breakdown, among the five methods, Tresca and Von Mises were more accurate than the other three, with Tresca being the most accurate of all.
3. All five methods displayed rotation as the most stressful movement for 1–8 mm of bone loss, while translation was the least stressful.
4. NVB was more exposed to ischemic risks than dental pulp during the periodontal breakdown due to constant tissular deformations during the 1–8 mm bone loss simulation, visible for all five movements.
5. Only VM and Tresca showed the translation movement as more prone to exposing dental pulp (both coronal and radicular) to more ischemic risks than the other five movements during the periodontal breakdown simulation.
6. All five methods showed intrusion and extrusion as more prone to exposing the NVB to higher ischemic risks than the other movements during the periodontal breakdown simulation.

## 6. Practical Implications

This research is the first to perform a comparison of five methods during horizontal periodontal breakdown and to highlight the methodological issues found in the current research flow with an impact on the current reports’ accuracy. It is also the first to study the biomechanical behavior of pulp and the NVB under 3 N of orthodontic force, during five movements in various levels of periodontal loss up to 8 mm loss. It is of clinically significant importance to know that 3 N of orthodontic force has almost no ischemic risks over the pulp and the NVB in healthy intact tissues, but this should be carefully used if previous injury/trauma are present in the conditions of bone loss presence. It also provides a complete set of biomechanical data extremely useful for the orthodontic planning phase. This study provides a clear comparative analysis, which is extremely okuseful when studying dental tissues. It also provides knowledge to better understand the nature of numerical studies as well as the requirements for accurate results.

## Figures and Tables

**Table 1:** Physical properties of materials.

Materials	Young’s Modulus, E (GPa)	Poisson Ratio, ʋ	Refs.
Enamel	80	0.33	[15,16,17,18,19,20,21,22]
Dentin/Cementum	18.6	0.31	[15,16,17,18,19,20,21,22]
Pulp and NVB	0.0021	0.45	[15,16,17,18,19,20,21,22]
PDL	0.0667	0.49	[15,16,17,18,19,20,21,22]
Cortical bone	14.5	0.323	[15,16,17,18,19,20,21,22]
Trabecular bone	1.37	0.3	[15,16,17,18,19,20,21,22]
Stainless streel bracket (Cr-Co)	218	0.33	[15,16,17,18,19,20,21,22]

**Table 2:** Maximum stress average values (KPa) produced by 3 N in the NVB and coronal pulp during periodontal breakdown and five orthodontic movements.

Resorption (mm)			1	2	3	4	5	6	7	8
Intrusion	**Tresca**	NVB	**0.84**	**0.99**	**1.13**	**1.28**	**1.50**	**1.73**	**1.95**	**2.17**
		c	**0.08**	**0.09**	**0.10**	**0.11**	**0.13**	**0.15**	**0.17**	**0.18**
	**VM**	NVB	**0.71**	**0.84**	**0.98**	**1.12**	**1.31**	**1.55**	**1.64**	**1.88**
		c	**0.07**	**0.08**	**0.09**	**0.10**	**0.11**	**0.13**	**0.15**	**0.16**
	Pressure	NVB	2.96	3.27	3.59	3.91	4.51	5.10	5.69	6.28
		c	0.27	0.31	0.35	0.40	0.58	0.77	0.96	1.14
	S1	NVB	−2.56	−2.90	−3.24	−3.57	−3.99	−4.40	−4.80	−5.22
		c	0.27	0.29	0.31	0.33	0.39	0.44	0.49	0.55
	S3	NVB	−2.99	−3.22	−3.45	−3.68	−4.17	−4.67	−5.16	−5.66
		c	0.20	0.20	0.21	0.21	0.27	0.32	0.38	0.44
Extrusion	**Tresca**	NVB	**0.84**	**0.99**	**1.13**	**1.28**	**1.50**	**1.73**	**1.95**	**2.17**
		c	**0.08**	**0.09**	**0.10**	**0.11**	**0.13**	**0.15**	**0.17**	**0.18**
	**VM**	NVB	**0.71**	**0.84**	**0.98**	**1.12**	**1.31**	**1.55**	**1.64**	**1.88**
		c	**0.07**	**0.08**	**0.09**	**0.10**	**0.11**	**0.13**	**0.15**	**0.16**
	Pressure	NVB	−2.96	−3.27	−3.59	−3.91	−4.51	−5.10	−5.69	−6.28
		c	−0.27	−0.31	−0.35	−0.40	−0.58	−0.77	−0.96	−1.14
	S1	NVB	2.98	3.44	3.90	4.35	4.69	5.03	5.36	5.70
		c	−0.55	−0.61	−0.67	0.72	0.74	0.77	0.81	0.83
	S3	NVB	3.05	3.29	3.52	3.75	4.29	4.82	5.36	5.89
		c	−0.26	−0.28	−0.30	−0.32	−0.36	−0.41	−0.44	−0.48
Translation	**Tresca**	NVB	**0.64**	**0.72**	**0.80**	**0.88**	**0.98**	**1.08**	**1.19**	**1.29**
		c	**0.11**	**0.12**	**0.14**	**0.15**	**0.16**	**0.17**	**0.17**	**0.18**
	**VM**	NVB	**0.55**	**0.62**	**0.69**	**0.76**	**0.86**	**0.96**	**1.07**	**1.17**
		c	**0.13**	**0.14**	**0.14**	**0.15**	**0.15**	**0.15**	**0.15**	**0.16**
	Pressure	NVB	2.43	2.54	2.65	2.76	3.23	3.71	4.19	4.66
		c	−0.35	−0.36	−0.37	0.38	0.45	0.51	0.58	0.64
	S1	NVB	−2.11	−2.24	−2.39	−2.51	−2.94	−3.36	−3.79	−4.22
		c	0.28	0.32	0.35	0.38	0.42	0.47	0.51	0.55
	S3	NVB	−2.57	−2.71	−2.85	−2.99	−3.52	−4.05	−4.58	−5.10
		c	−0.10	−0.11	−0.11	0.11	0.13	0.15	1.76	0.20
Rotation	**Tresca**	NVB	**0.90**	**1.21**	**1.51**	**2.07**	**2.32**	**2.57**	**2.82**	**3.07**
3 N		c	**0.16**	**0.17**	**0.18**	**0.19**	**0.21**	**0.24**	**0.26**	**0.28**
	**VM**	NVB	**0.97**	**1.19**	**1.41**	**1.63**	**1.74**	**1.85**	**1.95**	**2.06**
		c	**0.14**	**0.15**	**0.16**	**0.17**	**0.19**	**0.22**	**0.24**	**0.27**
	Pressure	NVB	3.98	4.53	5.08	5.63	6.33	7.03	7.73	8.42
		c	1.69	1.76	1.83	1.90	2.13	2.36	2.59	2.82
	S1	NVB	−3.98	−4.67	−5.33	−6.00	−6.72	−7.44	−8.16	−8.87
		c	0.88	1.20	1.51	1.83	2.08	2.32	2.57	2.81
	S3	NVB	−4.28	−4.95	−5.61	−6.28	−7.05	−7.83	−8.60	−9.37
		c	0.47	0.56	0.65	−0.74	−0.82	−0.91	−0.99	−1.08
Tipping	**Tresca**	NVB	**0.91**	**1.09**	**1.26**	**1.44**	**1.64**	**1.84**	**2.04**	**2.24**
		c	**0.13**	**0.14**	**0.14**	**0.14**	**0.16**	**0.17**	**0.18**	**0.19**
	**VM**	NVB	**0.79**	**0.94**	**1.10**	**1.25**	**1.43**	**1.62**	**1.80**	**1.98**
		c	**0.11**	**0.11**	**0.11**	**0.11**	**0.13**	**0.14**	**0.16**	**0.17**
	Pressure	NVB	3.09	3.55	4.01	4.47	4.94	5.42	5.89	6.36
		c	1.17	1.21	1.25	1.29	1.33	1.37	1.40	1.44
	S1	NVB	−2.80	−3.05	−3.29	−4.18	−4.57	−4.95	−5.34	−5.72
		c	−0.69	−0.88	−1.07	1.26	1.54	1.82	2.09	2.37
	S3	NVB	−3.19	−3.56	−3.92	−4.28	−4.87	−5.46	−6.05	−6.64
		c	−0.30	−0.35	−0.40	−0.44	0.45	0.47	0.47	0.48

NVB—neuro-vascular bundle, c—coronal pulp.

## Data Availability

The original contributions presented in this study are included in the article. Further inquiries can be directed to the corresponding author(s).
